# The Effect of Allopregnanolone on Enzymatic Activity of the DNA Base Excision Repair Pathway in the Sheep Hippocampus and Amygdala under Natural and Stressful Conditions

**DOI:** 10.3390/ijms21207762

**Published:** 2020-10-20

**Authors:** Tomasz Misztal, Paweł Kowalczyk, Patrycja Młotkowska, Elżbieta Marciniak

**Affiliations:** The Kielanowski Institute of Animal Physiology and Nutrition, Polish Academy of Sciences, Instytucka 3, 05-110 Jabłonna, Poland; p.kowalczyk@ifzz.pl (P.K.); p.mlotkowska@ifzz.pl (P.M.); e.marciniak@ifzz.pl (E.M.)

**Keywords:** allopregnanolone, stress, base excision repair, DNA glycosylases, hippocampus, amygdala

## Abstract

The neurosteroid allopregnanolone (AL) has many beneficial functions in the brain. This study tested the hypothesis that AL administered for three days into the third brain ventricle would affect the enzymatic activity of the DNA base excision repair (BER) pathway in the hippocampal CA1 and CA3 fields and the central amygdala in luteal-phase sheep under both natural and stressful conditions. Acute stressful stimuli, including isolation and partial movement restriction, were used on the last day of infusion. The results showed that stressful stimuli increased N-methylpurine DNA glycosylase (MPG), thymine DNA glycosylase (TDG), 8-oxoguanine glycosylase (OGG1), and AP-endonuclease 1 (APE1) mRNA expression, as well as repair activities for 1,*N^6^*-ethenoadenine (εA), 3,*N^4^*-ethenocytosine (εC), and 8-oxoguanine (8-oxoG) compared to controls. The stimulated events were lower in stressed and AL-treated sheep compared to sheep that were only stressed (except MPG mRNA expression in the CA1 and amygdala, as well as TDG mRNA expression in the CA1). AL alone reduced mRNA expression of all DNA repair enzymes (except TDG in the amygdala) relative to controls and other groups. DNA repair activities varied depending on the tissue—AL alone stimulated the excision of εA in the amygdala, εC in the CA3 and amygdala, and 8-oxoG in all tissues studied compared to controls. However, the excision efficiency of lesioned bases in the AL group was lower than in the stressed and stressed and AL-treated groups, with the exception of εA in the amygdala. In conclusion, the presented modulating effect of AL on the synthesis of BER pathway enzymes and their repair capacity, both under natural and stressful conditions, indicates another functional role of this neurosteroid in brain structures.

## 1. Introduction

Numerous free radicals, reactive oxygen species (ROS), and non-radical organic compounds containing functional groups are generated as by-products of natural respiration and metabolic processes in cells. In addition, the synthesis of reactive compounds can be induced by external factors, e.g., radiation, air pollutants, and industrial chemicals. They target and damage a wide range of macromolecules, including lipids, proteins, carbohydrates, and nucleic acids, and thereby impair cellular functions [1,2]. Among numerous biological lesions, DNA damage seems to be the most dangerous to life. By reacting with DNA, reactive molecules can cause single-base damage, strand breaks, as well as the formation of volume adducts [3]. 8-Oxoguanine (8-oxoG) is one of the most common types of oxidative base damage, with a high miscoding potential [4]. 1,*N^6^*-ethenoadenine (εA) and 3,*N^4^*-ethenocytosine (εC), are formed through reactions with lipid peroxidation products as etheno DNA adducts [5,6]. All of these base lesions are found in the DNA of untreated animals and humans, and can impair or deprive cells of the ability to correctly transcribe damaged DNA fragments or contribute to the fixation of potentially dangerous mutations of the cell’s genome [7,8].

In order to maintain the proper functioning of cells, various DNA repair mechanisms have evolved to prevent the accumulation of harmful mutations. These include the synthesis of antioxidant enzymes or enzymes that directly repair DNA damage. The primary and ubiquitous defense against lesions that do not heavily distort the DNA structure in mammalian cells is the base excision repair (BER) pathway, which is responsible for the removal of lesioned bases [3]. Repair is initiated by the action of a damage-specific DNA *N*-glycosylase that is responsible for the recognition and removal of an altered base [9,10]. Several DNA glycosylases with different substrate specificities have been identified in higher organisms, including mammals [10]. N-Methylpurine DNA glycosylase (MPG) excises εA from DNA and also repairs other DNA alkylation products, while thymine DNA glycosylase (TDG) excises εC and thymine [11]. 8-Oxoguanine glycosylase (OGG1) is the primary enzyme responsible for the excision of 8-oxoG, a product of hydroxyl radical interaction with guanine, as well as the substituted imidazole ring-opened purines [12]. The mentioned glycosylases cleave the N-glycosidic bond between the damaged base and the deoxyribose moiety, leaving behind an apurinic/apyrimidinic (AP) site. AP sites are then processed by assisting AP-endonuclease 1 (APE1), which may also act as a redox regulation enzyme for transcription factors [11].

Under normal physiological conditions, balance is maintained between endogenous oxidants and numerous enzymatic defenses. Disturbance of this balance may result in extensive oxidative damage to DNA, which in turn contributes to aging, malignant tumors, and other degenerative diseases [13]. Common acute and prolonged stressful stimuli can cause such disturbances, and some endogenous protective compounds can modulate their potency. Studies on rodents have shown that various acute stressors have stimulated the synthesis of allopregnanolone (AL) in the central nervous system (CNS), a neurosteroid, which in turn is able to protectively reduce neuroendocrine stress responses and mitigate their harmful effects [14,15,16]. The specific action of AL consists of enhancing the inhibitory effect of γ-aminobutyric acid (GABA) on the hypothalamic paraventricular nucleus (PVN), thereby shutting down the activity of the hypothalamic–pituitary–adrenal (HPA) axis [17]. On the contrary, chronic stress and related diseases were shown to reduce AL synthesis in the CNS and disrupt the mechanism that inhibits HPA axis activity [18,19,20]. An increasing amount of data show that treatment with AL or stimulation of its biosynthesis can be effective in restoring this GABAergic inhibition and alleviating adverse disease symptoms [21,22].

In this study, we tested the hypothesis that the beneficial effects of neurosteroids on the CNS could also affect DNA molecules, especially in the structures of the limbic system, such as the hippocampus and amygdala. Both of these structures are highly exposed to oxidative stress, while being involved in the regulation of stress responses, learning, and memory, as well as emotional behavior [23,24]. Therefore, we focused on the expression of genes encoding enzymes involved in the BER pathway (MPG, TDG, OGG1, and APE1) in two *cornu ammonis* (CA) fields of the hippocampus, namely CA1 and CA3, as well as in the central part of the amygdala of sheep under both natural and stressful conditions. Furthermore, repair activity of these enzymes was determined based on the excision efficiency of damaged nucleobases (ɛA, ɛC, and 8-oxoG) using the nicking method. The main goal was to investigate whether these processes were affected by AL administered directly to the CNS. We used the sheep, a large animal model whose brain has a high structural similarity to the human brain and is highly susceptible to stress [25].

## 2. Results

### 2.1. mRNA Abundance of DNA Repair Enzymes

**Figure 1:**
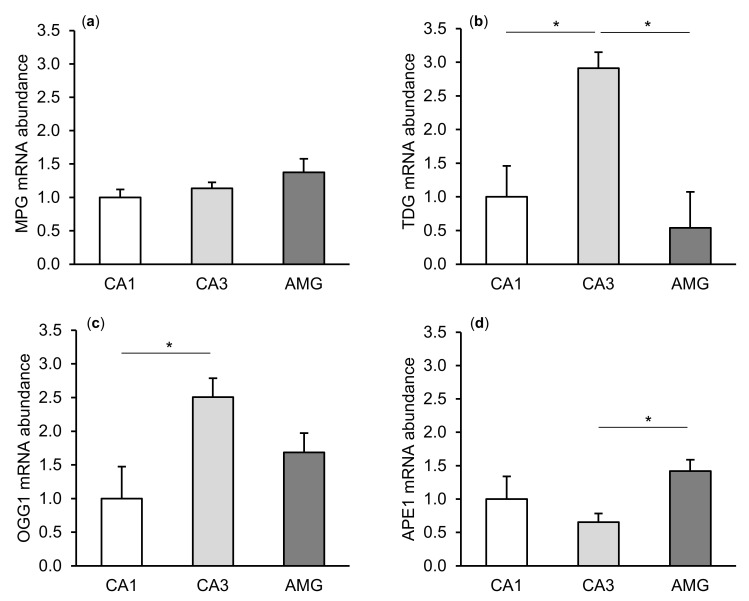
Relative mRNA abundance (mean ± SEM) of N-methylpurine-DNA glycosylase (MPG), (**a**) thymine-DNA glycosylase (TDG), (**b**) 8-Oxoguanine glycosylase (OGG1), (**c**) and AP-endonuclease 1 (APE1), (**d**) in the hippocampal CA1 and CA3 fields, as well as in the central amygdaloid nucleus (AMG) of the control group. Significance of differences: * *p* < 0.05.

The expression of gene transcripts for all enzymes of the DNA repair system (MPG, TDG, OGG1, and APE1) tested was found in both the CA1 and CA3 fields of the hippocampus, as well as in the central amygdaloid nucleus. The differences in the relative abundances of these transcripts between individual tissues of the control group are shown in Figure 1. Interestingly, a higher (*p* < 0.05) abundance of TDG and OGG1 mRNA was noted in the CA3 field of the hippocampus than in the CA1 field. The level of APE1 mRNA in the hippocampal CA3 field was, in turn, lower (*p* < 0.05) than in the amygdala.

**Figure 2:**
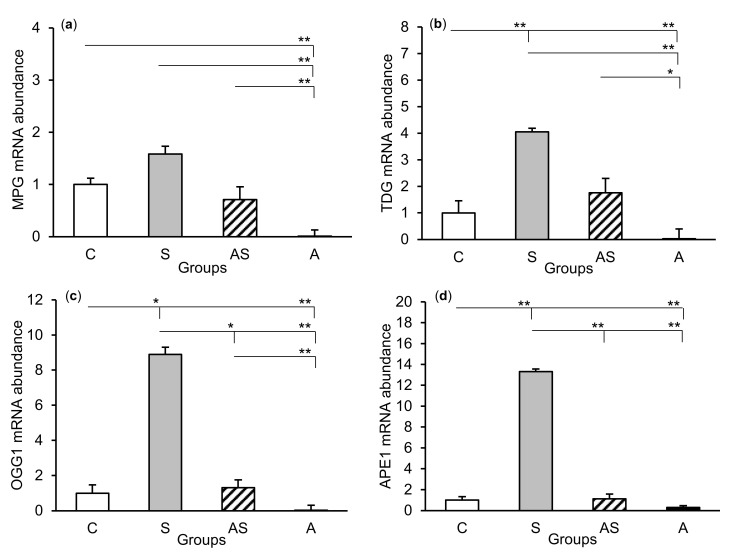
Relative mRNA abundance (mean ± SEM) of N-methylpurine-DNA glycosylase (MPG), (**a**) thymine-DNA glycosylase (TDG), (**b**) 8-oxoguanine glycosylase (OGG1), (**c**) and AP-endonuclease 1 (APE1), (**d**) in the hippocampal CA1 field of sheep treated with: vehicle (C), vehicle and stressful stimuli (S), allopregnanolone and stressful stimuli (AS), and allopregnanolone alone (A). Significance of differences: * *p* < 0.05, ** *p* < 0.01.

**Figure 3:**
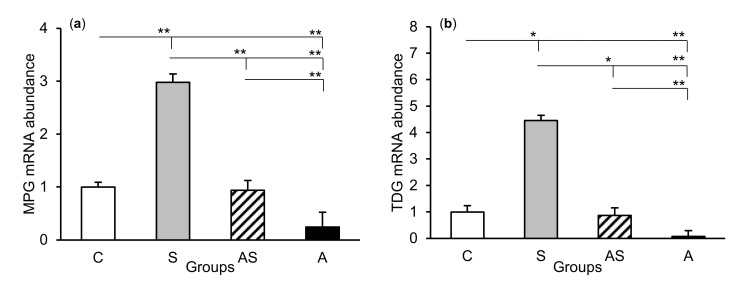

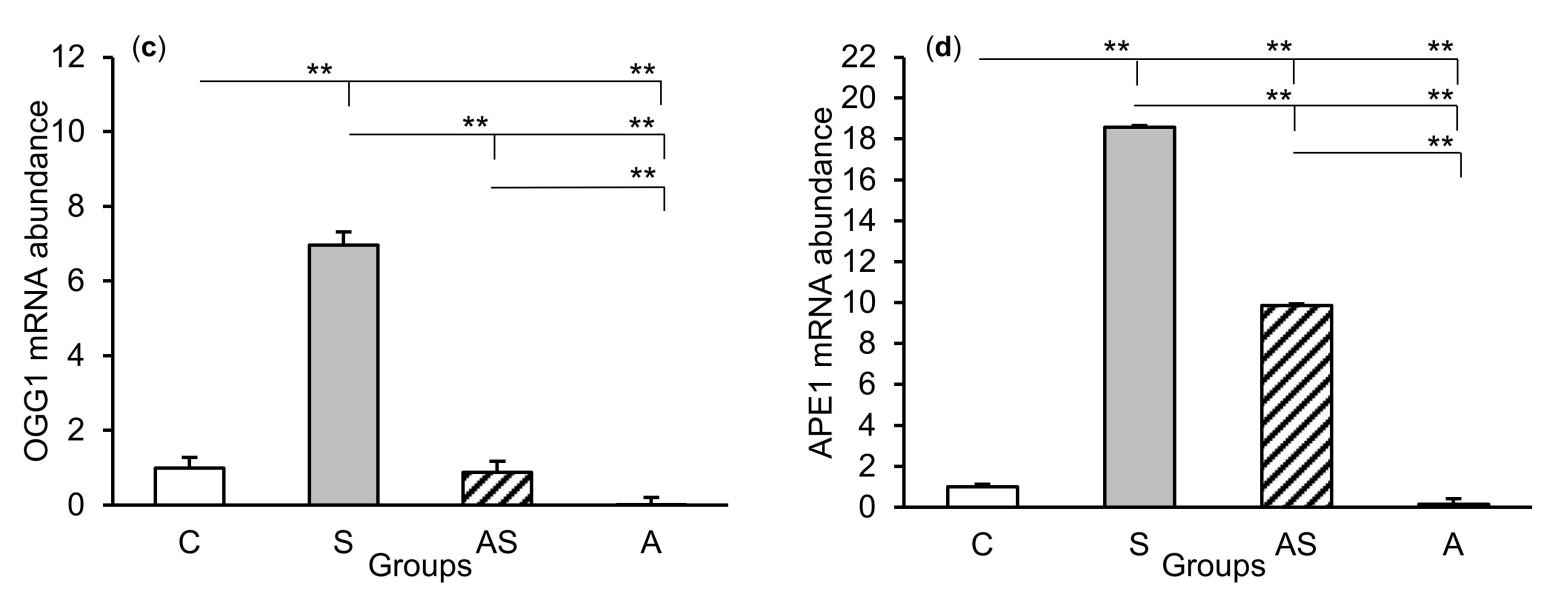
Relative mRNA abundance (mean ± SEM) of N-methylpurine-DNA glycosylase (MPG), (**a**) thymine-DNA glycosylase (TDG), (**b**) 8-oxoguanine glycosylase (OGG1), (**c**) and AP-endonuclease 1 (APE1), (**d**) in the hippocampal CA3 field of sheep treated with: vehicle (C), vehicle and stressful stimuli (S), allopregnanolone and stressful stimuli (AS), and allopregnanolone alone (A). Significance of differences: * *p* < 0.05, ** *p* < 0.01.

**Figure 4:**
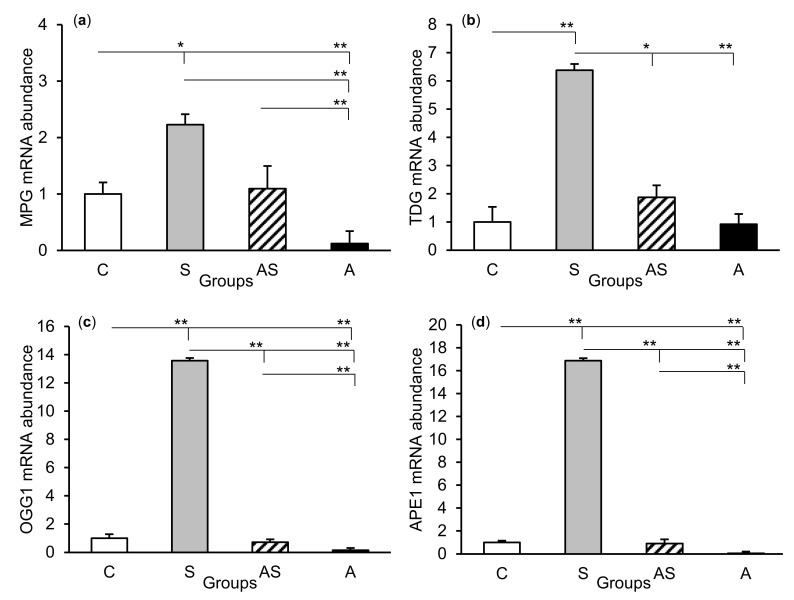
Relative mRNA abundance (mean ± SEM) of N-methylpurine-DNA glycosylase (MPG), (**a**) thymine-DNA glycosylase (TDG), (**b**) 8-oxoguanine glycosylase (OGG1), (**c**) and AP-endonuclease 1 (APE1), (**d**) in the central amygdaloid nucleus of sheep treated with: vehicle (C), vehicle and stressful stimuli (S), allopregnanolone and stressful stimuli (AS), and allopregnanolone alone (A). Significance of differences: * *p* < 0.05, ** *p* < 0.01.

The relative abundances of MPG, TDG, OGG1, and APE1 mRNA were compared among the treatment groups in all tissues studied, which are shown in Figure 2 (CA1), Figure 3 (CA3), and Figure 4 (central amygdala). In the S group, the abundances of TDG, OGG1, and APE1 mRNA increased in the CA1, CA3, and amygdala as compared to the control group (*p* < 0.05 – *p* < 0.01), while the abundance of MPG mRNA increased in the CA3 and amygdala (*p* < 0.05 – *p* < 0.01). Almost all mRNA stimulation cases were reduced in the AS group compared to the S group (*p* < 0.05 – *p* < 0.01), except MPG and TDG in the CA1 and MPG in the amygdala (despite the downward trend). The reduced transcript abundances were similar to controls and only the abundance of APE1 mRNA in the CA3 was still higher (*p* < 0.05) in the AS group than in the control. In the A group, the inhibitory effect of AL alone on the mRNA expression of all DNA repair enzymes predominated compared to the control group (*p* < 0.01, except TDG in the amygdala) and other groups (*p* < 0.05 – *p* < 0.01, expect APE1 in the CA1 and TDG in the amygdala vs. the AS group). A decrease in mRNA abundance in response to AL alone was in many cases up to the detection limits.

### 2.2. Enzyme Repair Activity

**Figure 5:**
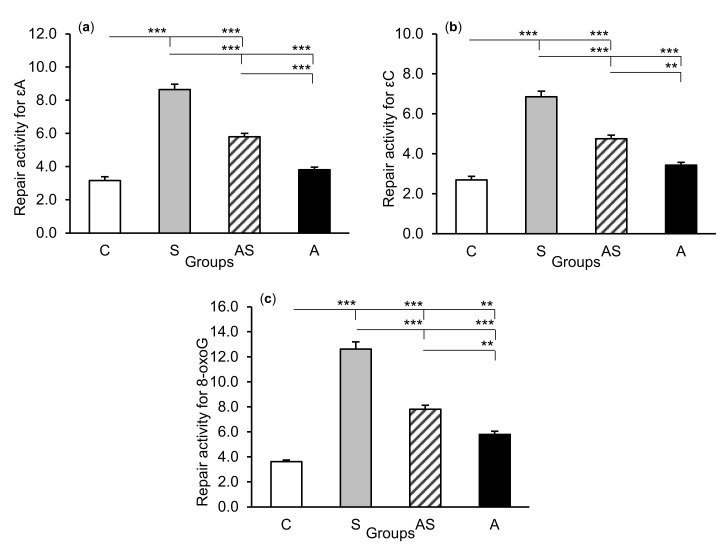
Repair activities (fmol/µg protein/h) for 1,*N^6^*-ethenoadenine (εA), (**a**) 3,*N^4^*-ethenocytosine (εC), (**b**) and 8-oxoguanine (8-oxoG), (**c**) in the hippocampal CA1 field of sheep treated with: vehicle (C), vehicle and stressful stimuli (S), allopregnanolone and stressful stimuli (AS), and allopregnanolone alone (A). Significance of differences: ** *p* < 0.01, *** *p* < 0.001.

**Figure 6:**
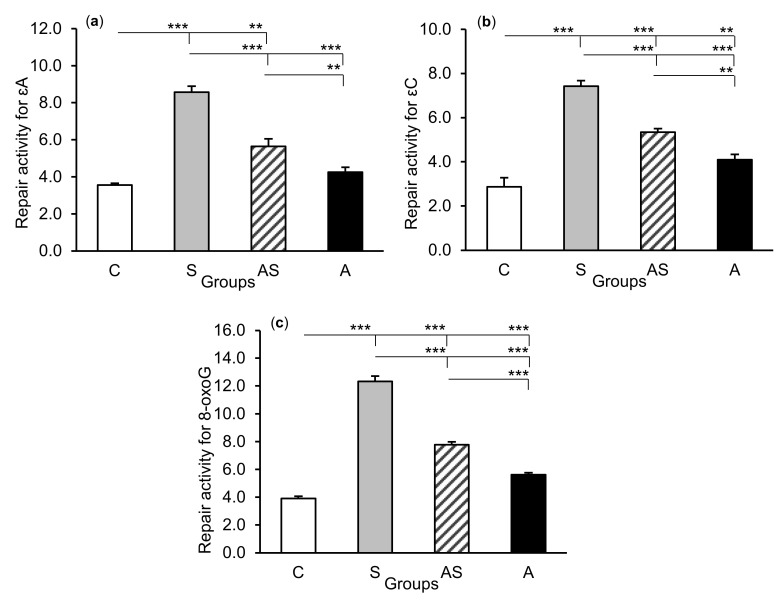
Repair activities (fmol/µg protein/h) for 1,*N^6^*-ethenoadenine (εA), (**a**) 3,*N^4^*-ethenocytosine (εC), (**b**) and 8-oxoguanine (8-oxoG), (**c**) in the hippocampal CA3 field of sheep treated with: vehicle (C), vehicle and stressful stimuli (S), allopregnanolone and stressful stimuli (AS), and allopregnanolone alone (A). Significance of differences: ** *p* < 0.01, *** *p* < 0.001.

**Figure 7:**
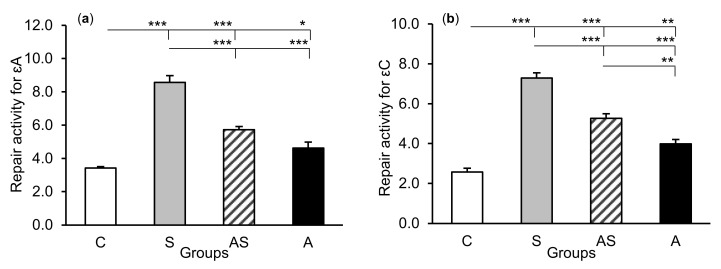

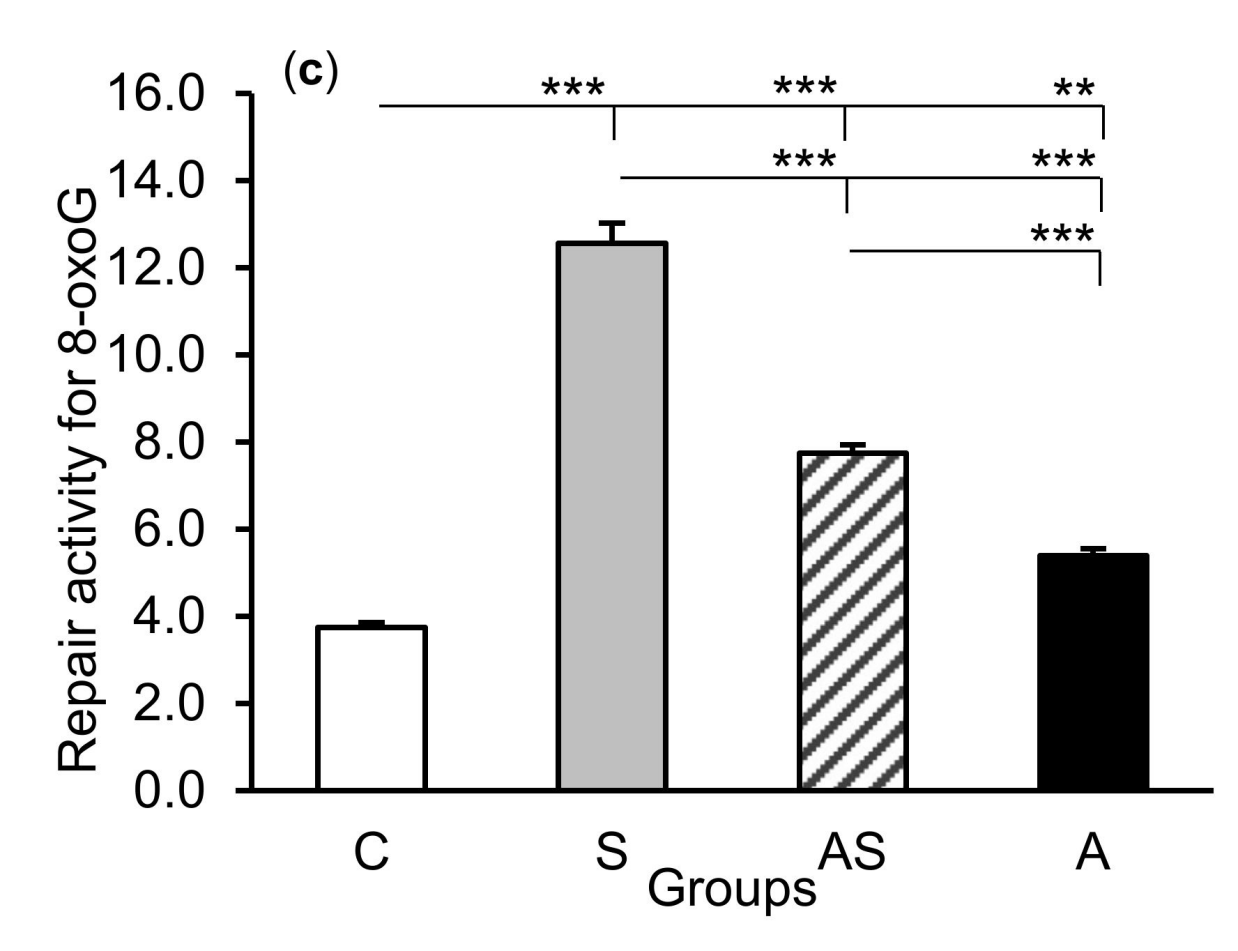
Repair activities (fmol/µg protein/h) for 1,*N^6^*-ethenoadenine (εA), (**a**) 3,*N^4^*-ethenocytosine (εC), (**b**) and 8-oxoguanine (8-oxoG), (**c**) in the central amygdaloid nucleus of sheep treated with: vehicle (C), vehicle and stressful stimuli (S), allopregnanolone and stressful stimuli (AS), and allopregnanolone alone (A). Significance of differences: * *p* < 0.05, ** *p* < 0.01, *** *p* < 0.001.

The exposure of sheep to stressful stimuli (S group) increased repair activities for εA, εC, and 8-oxoG compared to the control group (C group) in all tissues studied, i.e., in the CA1 (*p* < 0.001, Figure 5) and CA3 (*p* < 0.001, Figure 6) fields of the hippocampus, as well as in the central amygdaloid nucleus (*p* < 0.001, Figure 7), as measured by the nicking of an oligodeoxynucleotide with a single base modification. The abundance of excised εA, εC, and 8-oxoG decreased (*p* < 0.001) in the AS group in comparison with the S group, which was still higher (*p* < 0.001) than in the control sheep. The influence of AL alone (A group) on the DNA repair activities was varied and depended on the type of tissue. AL stimulated the excision of εA in the amygdala (*p* < 0.05), εC in the CA3 and amygdala (*p* < 0.01), and 8-oxoG in all tissues studied, including CA1 (*p* < 0.01), CA3 (*p* < 0.001), and amygdala (*p* < 0.01), as compared to controls. The rate of modified DNA base excision in the A group, however, was lower than in the S and AS groups (*p* < 0.01 – *p* < 0.001), with the exception of εA in the amygdala (vs. the AS group).

## 3. Discussion

This study documented the expression of DNA repair enzyme (*MPG, TDG, OGG1*, and *APE*) transcripts and the activity of these enzymes based on the efficiency of excision of damaged nucleobases (ɛA, ɛC, and 8-oxoG) in the sheep hippocampus and amygdala under both natural and stressful conditions. The principal finding of this study, however, was that centrally infused AL affected both of these processes, reducing DNA repair activities in stressed animals, as well as controlling the processing of DNA repair enzyme transcripts and nucleobase excision efficiency under natural conditions.

Both the hippocampus and amygdala are components of the limbic system. They process large amounts of information from various brain regions and are vital for learning and memory, as well as emotional behavior [23,24]. Moreover, the hippocampus is distinguished by numerous biochemical changes that ultimately determine neuronal connections and functions. It is also well known that the dentate gyrus–CA3 system exhibits structural plasticity with regenerative and remodeling capacity [26]. The chain of molecular events occurring within these structures has a high oxygen demand, leading to the exposure of neurons to the associated ROS byproducts [13]. It has been reported that the hippocampus and amygdala are among the brain structures that are most susceptible to oxidative stress and may succumb more easily to cell or DNA-damaging processes [27]. The results showed potential DNA protective and repair events concerning enzyme activities of the BER pathway, which take place in the sheep hippocampus and amygdala under normal conditions. It is worth noting the 2.5–3.0-fold lower abundances of *TDG* and *OGG1* transcripts in the hippocampal CA1 field than in the CA3 field, which is in agreement with the observation that hippocampal CA1 neurons are much more vulnerable than CA3 neurons to a variety of adverse conditions [28,29]; thus, they may provide greater post-transcriptional activities.

Each stressful stimulus recorded by the brain is transferred to the limbic system, as well as numerous signals and impulses detecting homeostasis disorders, [30,31]. The analysis and processing of stressful stimuli in the hippocampus and amygdala lead to the modification of the activity of other brain regions, including both the hypothalamic–pituitary–adrenal (HPA) axis and the adrenergic (sympathetic–adrenal) system [30,32]. Studies on adult rats have shown that in response to stress, the bed nucleus of the stria terminalis sends an axonal projection into neuroendocrine cell regions of the paraventricular nucleus (PVN) [33] and relays hippocampal cholinergic signals activating the HPA axis [34]. The results described in our earlier work [25] showed that the acute stressful stimuli used, i.e., isolation in combination with partial movement restriction, induced strong neuroendocrine stress response in experimental sheep. This was reflected by an increase in the concentration of corticotropin-releasing hormone (CRH) in the cerebrospinal fluid (CSF), as well as the concentration of adrenocorticotropic hormone (ACTH) and cortisol in the blood. In addition, increased abundance of the CRH and arginine vasopressin mRNA in the PVN was observed [25]. Considering more broadly the consequences of such types of stress at the molecular level, the results of this study showed the strong stimulation of DNA defense mechanisms in all tissues examined. This was reflected in an increase in the abundance of DNA repair enzyme mRNA, constituting the BER pathway. The extremely high (up to several dozen times higher) expression applied primarily to *OGG1* and *APE1* transcripts in stressed sheep could concern both the nerve and glial cells. Furthermore, mRNA expression of DNA repair enzymes was accompanied by the increased excision rate of lesioned nucleobases, as determined by the nicking assay, which could indicate increased DNA lesion formation and elevated levels of repair enzymes in the cells under oxidative stress. The highest repair activity was observed for 8-oxoG in all tissues examined. Such a difference may be due to the fact that APE1 can additionally potentiate the OGG1 turnover on damaged DNA, increasing the excision of 8-oxoG [35]. Further, 8-oxoG can also be repaired by nucleotide excision repair (NER), another major excision repair pathway, as shown in vivo in yeast and in an in vitro assay with mammalian cell-free extract containing NER proteins [36,37]. It may also be formed in the deoxynucleoside triphosphate pool, from which it could be incorporated to DNA [38]. Although the transcription of *MPG* and *TDG* genes and repair activity of the enzymes encoded by them in the tissues were not as pronounced in response to stressful stimuli as that of *OGG1* and *APE1*, they clearly suggested increased oxidative DNA damage derived from the adduction of lipid peroxidation products (εA, εC). It should be noted that the excised lesioned bases could be of both nucleic and mitochondrial origin, because both of these cell organelles are the sites of oxidative DNA damage and active DNA repair pathways [39]. The results of other studies have shown that the induction of immunological stress in rats through treatment with lipopolysaccharide increased enzyme expression and repair activities in a similar way [40]. Accumulating evidence links oxidative stress, associated with high DNA repair enzyme mRNA levels, with the progression and complications of many diseases, including the carcinogenesis process and autoimmunity [7,41,42]. Moreover, 8-oxoG has been established as an important biomarker of oxidative stress [12,43], cancer risk [44,45], and ageing processes, including degenerative diseases [46,47], and in general as a biological marker of lifestyle and the effects of diet [48,49].

The problem of whether the transmitted stress signals in and of themselves impair neuronal activity or whether this is mediated by adrenal corticosteroids, which are the final effectors of the HPA axis, still persists. The neurotransmitter systems that underlie hippocampal changes during stress episodes are still not entirely elucidated, but likely involve several neurotransmitters, such as excitatory amino acids, serotonin and γ-aminobutyric acid (GABA). All these compounds may cause disturbances in the functioning of the hippocampus cells, also contributing to their damage [50,51,52]. It has been shown that increased glutamate release may initiate excitotoxicity, which consists of the denaturation of a variety of lipids and proteins and DNA damage, leading to neuronal death [53]. On the other hand, both glucocorticoids and mineralocorticoids, acting through their dual-receptor system localized in limbic areas, function coordinately in controlling stress reactions, but also have a negative impact on affective and cognitive processes [54,55,56]. Several studies on rodents and monkeys, as well as preclinical investigations, have indicated hippocampal structural damage following exposure to high glucocorticoid levels or various stressors [57,58,59]. Numerous described cases could have been preceded by damage at the molecular level, whose common feature was oxidative stress.

The synthesis of neurosteroids occurs in nerve cells in various brain regions, including glutamatergic pyramidal neurons in the cortex, hippocampus, and the basolateral amygdala [60,61]. Previous studies on rats and mice indicated that acute stressors such as swim stress, CO_2_ inhalation, and LPS treatment were associated with increases in neuroactive steroid concentrations in the plasma and brain [14,62,63,64]. Purdy et al. [14] showed that the AL concentration in the rat brain (cerebral cortex and hypothalamus) increased for an hour after stress initiation, prior to peaking in the plasma. Moreover, Concas et al. [65] suggested that the rate of AP synthesis in the brain was greater than in peripheral blood-supplying tissues. By examining earlier the central effect of AL on HPA axis activity in the same sheep, we found that this neurosteroid reduced numerous aspects of neuroendocrine stress response when administered into the IIIv [25]. This was reflected, among other factors, by a decrease in the concentration of CRH in the CSF, as well as of ACTH and cortisol in plasma. In this aspect, AL was shown to enhance GABA-mediated inhibitory action on PVN neurons by interacting with the membrane-bound GABA_A_ receptors [66]. A protective action of AL against stress hormones, as well as its other beneficial effects on the CNS, have been widely documented in rodents and humans [64]. The presented research demonstrated that the protective effect of AL could also involve a DNA defense mechanism. As shown, both transcript expression of DNA repair enzymes and the efficiency of damaged base excision decreased in animals treated for three days with AL and exposed to stressful stimuli (group AS vs. group S). This suggested that AL could contribute to reducing DNA damage, as well as inhibiting stress-induced hyperactivity of enzymes to protect the stability of DNA molecules, by counteracting over-induced mechanisms. It is also possible that the cells accumulated a certain pool of DNA repair enzymes as a result of AL infusions preceding stress. According to this, in several cases we simultaneously observed reduced mRNA expression of repair enzymes and increased excision efficiency of damaged bases after AL treatment alone compared to the control group. It is unlikely that this neurosteroid induced DNA damage by itself, because it could increase the number of lesions in stressed animals. Thus, AL alone could rather increase post-transcriptional or translational processing of repair enzyme mRNAs. In addition, diverse expression of DNA repair enzyme transcripts and inconsistent response to stress and AL in individual tissues could indicate their different vulnerability to such challenges.

To our knowledge, there are no data concerning the direct or indirect effects of AL on enzymatic DNA protection mechanisms. It is possible that the effect of AL on DNA repair is related to the level of brain-derived neurotrophic factor (BDNF), since BDNF has been described as increasing neuronal survival, at least in part by inducing transcription of DNA repair enzymes such as APE1 [67]. Increasing amounts of data reveal positive relationships between AL and BDNF and point to the hippocampus as the main region of neurotrophic regulations mediated by this neurosteroid [68]. In other studies, Cho et al. [69] showed that AL could have a neuroprotective antioxidant effect in the hippocampus of mice by increasing both superoxide dismutase (SOD; antioxidant enzyme) expression and its functional activity. Moreover, AL reduced the levels of ROS and lipid peroxidation in human Niemann–Pick C fibroblasts [70], as well as promoted SOD activity and decreased cell death in the Alzheimer’s disease model of PC12 cells [71]. In light of the above disease models, in which AL has been shown as a potential therapeutic agent, our study sheds new light on its protective function in the CNS with respect to the DNA repair process in conditions of disturbed homeostasis.

In conclusion, acute stressful stimuli induced enzymatic activity of the BER pathway in the sheep hippocampus and amygdala. The presented modulating effect of AL on the synthesis of the BER pathway enzymes and their repair capacity, both under natural and stressful conditions, indicates another functional role of this neurosteroid in brain structures.

## 4. Materials and Methods

### 4.1. Animal Management

All animal procedures were conducted in accordance with the Polish Act on the Protection of Animals Used for Scientific or Educational Purposes (2015) and were approved by the Second Local Ethics Committee for Animal Experiments, Warsaw University of Life Sciences—SGGW, Warsaw, Poland (Resolution No. WAW2-24/2016). Twenty-four Polish Longwool sheep (a breed representing reproduction seasonality) aged two years and weighing 55 ± 3 kg were used in the experiment. They were farmed at the Sheep Breeding Center of the Kielanowski Institute of Animal Physiology and Nutrition, Polish Academy of Sciences. The animals were kept indoors in individual pens under natural lighting conditions (52°N, 21°E) and fed twice a day with a diet based on pelleted concentrate and hay according to the recommendations of the National Research Institute of Animal Production (Krakow–Balice, Poland)—National Institute for Agricultural Research (France) [72]. All sheep had free access to water and mineral licks.

### 4.2. Experimental Design and Brain Tissue Collection

Tissue material samples were collected from sheep, for which the effect of centrally administered allopregnanolone on the neuroendocrine response to acute stressful stimuli was described in a previous work [22]. More specifically, sheep were implanted with a stainless steel guide cannula into the third brain ventricle (IIIv) (outer diameter 1.2 mm, positions: frontal 30.5–31.5 mm and sagittal 1.0 mm) in accordance with the stereotaxic coordinate system for the sheep hypothalamus [73]. The implantation was performed under general anesthesia (xylazine: 40 mg/kg of body mass, intravenously; xylapan and ketamine: 10 to 20 mg/kg of body mass, intravenously; Bioketan, Vetoquinol Biowet, Pulawy, Poland) through a hole drilled in the skull, in accordance with the procedure described by Traczyk and Przekop [74]. The guide cannula was fixed to the skull with stainless steel screws and dental cement. The external opening of the canal was closed with a stainless steel cap. After the surgery, ewes were injected daily with antibiotics for 5 d (1 g streptomycin and 1,200,000 IU benzylpenicillin; Polfa, Warsaw, Poland) and with analgesics for 4 d (metamizole sodium 50 mg/animal; Biovetalgin, Biowet Drwalew, Drwalew, Poland, or meloxicam 1.5 mg/animal; Metacam, Boehringer Ingelheim, Ingelheim am Rhein, Germany). Placement of the cannula into the IIIv was confirmed by CSF outflow during surgery and after slaughtering.

The experiment was performed during the reproductive season from mid-October to mid-December. Estrus synchronization was performed using the Chronogest CR method [25]. The sheep were randomly divided into four groups (*n* = 6 each) and were treated over three consecutive days of the late luteal phase (days 12–14) of the estrous cycle as follows: (1) intracerebroventricular (icv.) infusion of a vehicle for three days (C group); (2) icv. infusion of a vehicle and use of stressful stimuli on the third day (S group); (3) icv. infusion of AL and use of stressful stimuli on the third day (AS group); (4) icv. infusion of AL alone for three days (A group). All infusions were performed in a series of four 30 min infusions at 30 min intervals (from 10:00 to 14:00 h). The AL dose, 4 × 15 μg/60 μL/30 min/day, was selected on the basis of our preliminary study (unpublished data, Grant No. 2015/19/B/NZ9/03706). A BAS Bee microinjection pump (Bioanalytical Systems Inc., West Lafayette, IN, USA) and calibrated 1.0-mL gas-tight syringes were used. During the treatments, sheep were kept in pairs in the experimental room in comfortable cages, where they could lie down and to which they were previously adapted. Stressful stimuli included isolation (removal of untreated sheep) and partial movement restriction (protection against escaping from the cage). Their duration covered the period of infusions (4 h).

Immediately after the experiment, sheep were slaughtered after prior pharmacological stunning (xylazine 0.2 mg/kg of body mass and ketamine: 3 mg/kg of body mass, intravenously) and the brains were rapidly removed from the skull. After separation of the median eminence and cerebellum, each brain was sectioned sagittally into the cerebral hemispheres. The hippocampus was dissected from the medial part of the temporal lobe of the right hemisphere, starting from the floor of the lateral ventricle through ventral and dorsal parts, according to the sheep brain atlas [75]. Sections measuring 2–3 mm in length were cut out from the CA1 and CA3 regions of the hippocampus. Subsequently, a block of the right amygdala (thought to be linked to negative emotions [76]) covering the central amygdaloid nucleus (thought to be involved in behavioral, autonomic, and endocrine responses [77]) was isolated by a point cut-out to a depth of approximately 2 mm. All tissue cuts were performed on sterile glass plates placed on ice, then the collected structures were immediately frozen in liquid nitrogen and stored at −80 °C.

### 4.3. Drug Preparation

A day before infusion, AL (Sigma-Aldrich, Saint Louis, MO, USA) was dissolved in a mixture (1:1) of dimethyl sulfoxide (DMSO) (Blirt, DNA-Gdańsk, Gdańsk, Poland) and 20% 2-hydroxypropyl-β-cyclodextrin (Sigma-Aldrich, Saint Louis, MO, USA). After 24 h, the mixture was diluted in a Ringer–Locke (RL) solution and divided into portions for individual sheep. The mixture without AL was diluted in RL and used as a control vehicle. Each portion of the infusion mixture contained 2% DMSO.

### 4.4. Relative Abundance of mRNA Analysis

Total RNA from the hypothalamic and hippocampal tissues was isolated using the NucleoSpin RNA II kit (Machery-Nagel GmbH and Co., Düren, Germany) at 4 × 4500, according to the manufacturer’s protocol. The concentration and purity of isolated RNA were quantified using a NanoDrop ND-1000 spectrophotometer (Thermo Fisher Scientific, Waltham, MA, USA). RNA integrity was electrophoretically verified using 1.5% agarose gel stained with ethidium bromide. Total RNA from each sample was used to generate cDNA using an Advantage RT-for-PCR cDNA synthesis kit (Clontech Laboratories Inc., Mountain View, CA, USA) with oligo (dT) primers, according to the manufacturer’s protocol. Briefly, 1 µg of total RNA was used as a starting material, to which we added 1 µl of oligo (dT), 1 µl of 10 mM dNTPs, 4 µl of 5 × reaction buffer, and 0.5 µl of recombinant RNAse inhibitor. The RNA and oligo (dT) reagents were mixed first. Then, we heated the contents at 70 °C for 2 min and chilled them on ice until the other components were added. Then, 1 µl of MMLV Reverse Transcriptase (20 U/u) was added and the samples were incubated at 42 °C for 60 min. The reaction was inactivated at 94 °C for 5 min.

Real-time PCR assays were carried out on the Applied Biosystems 7500 apparatus. Each single measurement was carried out in 25 μL reaction mixture containing: 1 × concentrated commercial buffer (without MgCl_2_) supplied with Taq polymerase; 3 mM MgCl_2_; 0.01% Tween 20; 0.8% glycerol; 5% DMSO; 0.5 ng/μL acetylated BSA; dATP, dCTP, dGTP, and dTTP at 400 μM each; 1 × concentrated reference dye (ROX); 1:40,000 diluted SYBR Green dye; 0.625 U of Taq polymerase, forward and reverse primers at 400 μM each; and an appropriate amount of template cDNA. The time–temperature program was as follows: 95 °C for 3 min as the initial denaturation step, followed by 45 cycles consisting of the denaturation step at 95 °C for 30 s, primer annealing at 60 °C for 30 s, and the extension step at 72 °C for 1 min. Fluorescence was read during the extension step of each cycle. Melting point temperature analysis was performed in the range of 60 °C to 92 °C with temperature increments of 0.33 °C. The background range and threshold for C_t_ evaluation in each experiment were adjusted manually.

All primers were designed using the Primer Express program (Applied Biosystems, Foster City, CA, USA) and their sequences are shown in Table 1. The product from each pair was 115–140 bp long. Prior to use, the primers were verified for equal efficiency of the PCR reaction. In order to ensure that the 2^-ΔΔCt^ validation method was applied [78], each experiment involved measurement of c_t_ values for four or five amounts of the template, each in duplicate. The template amounts per sample were 10, 40, 160, and 640 ng. During measurement of mRNA expression, for each cDNA sample four reactions were carried out using two template amounts, 160 and 640 ng, each in duplicate. The quality of the results was evaluated based on the expected c_t_ differences between two cDNA amounts, as well as product melting curves. Rare outlying results were omitted in calculations. For each gene, the amounts of cDNA used were chosen individually (if possible, the same for all genes) in order to obtain c_t_ values in the range of 14 to 34 cycles [40]. All c_t_ values were normalized to higher cDNA amounts used for the reference gene, then the standard ΔΔc_t_ method was applied to evaluate ratios between the expression of each measured gene vs. the reference gene (*18S*).

### 4.5. Enzyme Repair Activity

The oligonucleotides (40-mer) containing a single 1,*N*^6^-ethenodeoxyadenosine (εA), 3,*N*^4^-ethenodeoxycytosine (εC), or 8-oxo-guanine (8-oxoG) at position 20 in the sequence 5′-d(GCT ACC TAC CTA GCG ACC TXC GAC TGT CCC ACT GCT CGA)-3′, where X indicates εA, εC, or 8-oxoG, were obtained from Eurogentec Herstal (Herstal, Belgium) or Genset Oligos (Paris, France).

The oligonucleotides were ^32^P-labeled at the 5′-end by polynucleotide kinase at an excess of [γ^32^P]ATP (3000 Ci/mmol) (Amersham, Little Chalfont, UK). Radiolabeled oligomers were purified from unincorporated radioactivity using Micro Bio-Spin P-30 columns, as described by the manufacturer (Bio-Rad, Hercules, CA, USA). These oligomers were annealed at double-molar excess to complementary oligonucleotides containing T opposite εA, G opposite εC, or C opposite 8-oxoG. Complementary oligodeoxynucleotides were synthesized according to standard procedures using an Applied Biosystems synthesizer (Oligonucleotide Synthesis Laboratory, Institute of Biochemistry, and Biophysics, Polish Academy of Sciences).

The procedures for the repair activity assay were described in detail by Kowalczyk et al. [40]. In brief, tissues were homogenized with 4 volumes of buffer containing 50 mM Tris-HCl (pH 7.5), 1 mM EDTA, 10 mM DTT, and 1 mM phenylmethylsulfonyl fluoride. Cells were disrupted by sonification in the “high” mode of a Bioruptor sonicator (Diagenode, Denville, NJ, USA) (three 15 s pulses at 30 s intervals). Cell debris was removed by centrifugation (7000× *g*, 4 °C, 30 min) and the supernatant served as a source of enzymes. Protein concentration was determined using Bradford’s method [79]. Tissue repair activities for εA, εC, and 8-oxoG were measured by nicking the oligodeoxynucleotides at the site of the lesion in the tissue extract, which supplied both DNA glycosylases and AP-endonuclease. The reaction mixture at a total volume of 20 μL contained 25 mM Tris-HCl (pH 7.8), 50 mM NaCl, 5 mM b-mercaptoethanol, 1 mM EDTA, 1 pmol 32P-labeled duplex, and increasing amounts of tissue extract (1–100 μg protein/sample). Samples were incubated at 37 °C for 1 h and reactions were stopped by digestion with proteinase K (1 μg/μL of reaction mixture, 1 h, 37 °C). Since the lyase activity of the OGG1 protein is approximately 5- to 10-fold lower than that of glycosylase and the enzyme strongly binds to the AP site created after 8-oxoG excision, the reaction mixtures for 8-oxoG excision activity were incubated in 0.2 M NaOH at 70 °C for an additional 30 min. This incubation allowed complete cleavage of the oligonucleotide at the AP site formed after 8-oxoG excision. The reaction products were separated on 20% polyacrylamide gels with 7M urea as a denaturing factor. Electrophoresis was carried out in 1 × concentrated TBE buffer for 2–3 h at 400–600 V using a CONSORT 3000 v–300 mA high-voltage power supply. A Storm PhosphorImager was used for detection and ImageQuant software (GE Healthcare, Chicago, IL, USA) was applied to quantify reaction products. All estimations were performed at least in triplicate and are presented as femtomoles of cleaved 32P-oligo per hour per microgram of protein. In each experiment, two control samples were used: (a) a negative control in which a non-treated oligonucleotide was subjected to denaturing PAGE to show any possible degradation that could appear during the procedure, and (b) a positive control in which a labelled oligonucleotide was digested with an excess of the appropriate pure DNA glycosylase and human AP-endonuclease for ɛA and ɛC (gifted by Dr Murat Saparbaev and Dr Jacques Laval, Institut Gustave Roussy, Villejuif, France).

### 4.6. Statistical Analysis

Initially, all data were tested for normality with the Shapiro–Wilk normality test and then grouped into parametric and non-parametric groups. Tissue concentrations of excised damaged bases were analyzed using one-way analysis of variance (ANOVA) (STATISTICA, Stat Soft, Tulsa, OK, USA). Each analysis was followed by the post hoc least significant difference test. Statistical evaluations of differences in MPG, TDG, OGG1, and APE1 mRNA expression in the hippocampus and amygdala between treatment groups were carried out using non-parametric statistics, involving the Kruskal–Wallis test, followed by multiple comparisons of average ranks, then the Mann–Whitney U-test for particular groups. Differences were considered significant at *p* < 0.05, and all data are presented as a mean ± standard error of the mean (SEM).

## Figures and Tables

**Table 1:** Sequences of specific primers.

Gene	Primers (5′–3′)	GenBank Accession Number	Amplicon Size
*MPG*	F: GCTGAGGGCCAGCCAACACCTGC R: CGCCCCTTTACCCACGGAGCCCA	NC_040275.1/XM027962018.1	140
*TDG*	F: ACACAGGATGCTGTGGGGCT R: TCCCTCGGCCTAGAATTTTC	NC_040254.1	120
*OGG1*	F: CAGTCATAATAACAGTA R: AACCTCCTCTAAGCACTCAT	NC_040270.1/XM004018285	140
*APE1*	F: TTAGACATTTGGTTGCC R: GGCACCAACAGGGCTAGCA	NC_040272.1	140
*18S rRNA*	F: GCAATTATTCCCCCATGAACG R: GGGACTTAATCAACGCAAGC	NR_003286	115

MPG: N-Methylpurine DNA glycosylase, TDG: Thymine DNA glycosylase, OGG1: 8-Oxoguanine glycosylase, APE1: AP-endonuclease 1, 18S rRNA: 18S ribosomal RNA, F: forward primer, R: reverse primer. The real-time PCR amplification efficiencies of target and reference genes were in the range of 96–101%.

## Abbreviations

εA	1,*N^6^*-ethenoadenine
εC	3,*N^4^*-ethenocytosine
8-oxoG	8-oxoguanine
IIIv	Third brain ventricle
ACTH	Adrenocorticotropic hormone
AL	Allopregnanolone
AMG	Amygdaloid nucleus
ANOVA	Analysis of variance
AP	Apurinic/apyrimidinic site
APE1	AP-endonuclease 1
BDNF	Brain-derived neurotrophic factor
BER	Base excision repair
CA	*Cornu ammonis*
CNS	Central nervous system
CRH	Corticotropin-releasing hormone
CSF	Cerebrospinal fluid
DMSO	Dimethyl sulfoxide
GABA	γ-aminobutyric acid
HPA	Hypothalamic–pituitary–adrenal axis
icv	Intracerebroventricular
MPG	N-methylpurine DNA glycosylase
NER	Nucleotide excision repair
TDG	Thymine DNA glycosylase
OGG1	8-oxoguanine glycosylase
PVN	Paraventricular nucleus
RL	Ringer-Locke solution
ROS	Reactive oxygen species
SEM	Standard error of the mean
SOD	Superoxide dismutase
